# Pet Play and Socialization in Aging Communities: Exploring Engagement, Satisfaction, and Age Differences

**DOI:** 10.1093/geroni/igaf122.3640

**Published:** 2025-12-31

**Authors:** Erreannau Zellous, Taylor Pope

**Affiliations:** Miami University, Oxford, Ohio, United States; Purdue University, West Lafayette, Indiana, United States

## Abstract

Animal companionship among older adults, including therapy and pet ownership, has been associated with improved socialization and well-being. Older adults often face a heightened risk of social isolation and loneliness, with pet interactions potentially facilitating social connection and companionship. While research links the general benefits of pet ownership, fewer studies examine how active pet engagement, such as play, relates to socialization in later life. Guided by Socioemotional Selectivity Theory, pet play may align with emotional goals by fostering satisfying interpersonal exchanges. The Biophilia Hypothesis further supports this connection, by framing pet play as a natural and rewarding form of human connection. Using data from the General Social Survey (GSS, 2018; N = 239, aged 55+), an ordinal logistic regression model examined the association between hours spent playing with pets and social satisfaction, controlling for demographic and socioeconomic factors. Pet play was positively associated with higher social satisfaction (β = 0.16, p = 0.01). To explore age differences, a second set of regressions was estimated, stratified by age group (ages 55–64, 65–74, and 75+). This analysis used the same independent, dependent, and control variables within each age subgroup (significant positive association for ages 55–64: β = 0.56, p = .005; nonsignificant for ages 65–74: β = 0.35, p = .122, and ages 75+: β = 0.11, p = .736). Findings suggest that pet play may support socialization among older adults, particularly the youngest-old. Results have implications for aging services and future research exploring pet play’s benefits across the lifespan.

